# Gay fathers’ motivations for and feelings about surrogacy as a path to parenthood

**DOI:** 10.1093/humrep/dex026

**Published:** 2017-02-23

**Authors:** L. Blake, N. Carone, E. Raffanello, Jenna Slutsky, A. A. Ehrhardt, S. Golombok

**Affiliations:** 1 Centre for Family Research, University of Cambridge, Free School Lane, Cambridge CB2 3RF, UK; 2 Department of Developmental and Social Psychology, Sapienza University of Rome, Via dei Marsi 78, 00185 Rome, Italy; 3 Division of Gender, Sexuality, and Health, New York State Psychiatric Institute, Columbia University, 1051 Riverside Drive New York, NY 10032, USA

**Keywords:** surrogacy, gay father, genetic relatedness, same-sex couples, gestational surrogacy, genetic surrogacy

## Abstract

**STUDY QUESTION:**

Why do gay men choose to start their families through surrogacy?

**SUMMARY ANSWER:**

Most fathers chose surrogacy because they considered adoption to be a less desirable and/or accessible path to parenthood.

**WHAT IS KNOWN ALREADY:**

Little is known of gay fathers’ motivations to use surrogacy as a path to parenthood over and above other forms of family building, such as adoption, and no studies have examined fathers’ satisfaction with the surrogacy process.

**STUDY DESIGN, SIZE, DURATION:**

This study used a cross-sectional design as part of a larger investigation of parent–child relationships and child adjustment in 40 gay father surrogacy families. Multiple strategies (e.g. surrogacy agencies, social events and snowballing) were used to recruit as diverse a sample as possible. Data were obtained from 74 fathers (in 6 families only 1 father was available for interview).

**PARTICIPANTS/MATERIALS, SETTING, METHOD:**

Semi-structured interviews, lasting ~1 h, were conducted in the family home (65%) or over Skype (35%) with 74 gay fathers (35 genetic fathers, 32 non-genetic fathers and 7 fathers who did not know or did not disclose who the genetic father was), when the children were 3–9 years old.

**MAIN RESULTS AND THE ROLE OF CHANCE:**

Genetic and non-genetic fathers were just as likely to want to become parents and had similar motivations for choosing surrogacy as a path to parenthood. Most fathers (*N* = 55, 74%) were satisfied with surrogacy and were satisfied (*N* = 31. 42%) or had neutral feelings (*N* = 21, 28%) about their choice of who would be the genetic father. Most fathers received supportive reactions to their decision to use surrogacy from both families of origin (e.g. parents, siblings) (*N* = 47, 64%) and from friends (*N* = 63, 85%).

**LIMITATIONS, REASONS FOR CAUTION:**

Although diverse recruitment strategies were used, data were obtained from a volunteer sample. Therefore, the possibility that fathers who had a positive surrogacy experience may have been more likely to participate in the study, and therefore introduce bias, cannot be ruled out. Due to the high average annual income of the fathers in the study, findings may not generalize to gay fathers with lower incomes.

**WIDER IMPLICATIONS OF THE FINDINGS:**

It is often assumed that parents’ primary motivation for using ART is to have a genetic connection to the child. This study revealed that whilst genetic fatherhood was important for some gay fathers in surrogacy families, it was not important for all. This information will be of use to surrogacy agencies and organizations supporting men who are considering the different routes to parenthood.

**STUDY FUNDING/COMPETING INTEREST(S):**

This work was funded by the Wellcome Trust [097857/Z/11/Z] and the Jacob's Foundation. None of the authors has any conflict of interest to declare.

**TRIAL REGISTRATION NUMBER:**

N/A.

## Introduction

In 2014, ~37 800 male same-sex couples were raising children in the USA (US Census Bureau, 2014). Gay couples who wish to become parents may do so through surrogacy, a process in which a woman carries a pregnancy to term with the intention to relinquish the child to the intending parent(s). Intending fathers may choose to conceive using gestational surrogacy, where an embryo is created using the sperm of one partner and the egg of a donor, which is then transferred to the surrogate, or genetic surrogacy, in which conception occurs using the sperm of one partner and the egg of the surrogate.

Little is known about the motivations of gay couples who pursue surrogacy as a path to parenthood. However, several studies have explored the motivations of gay men who decide to adopt (Goldberg *et al.,* 2012; Jennings *et al*., 2014). Most gay and lesbian adoptive parents choose adoption as their preferred route to parenthood, in contrast to heterosexual couples who typically consider adoption only after failed attempts at natural and/or assisted reproduction (Jennings *et al*., 2014). In this UK study (Jennings *et al*., 2014), gay adoptive fathers were uncomfortable with the moral issues and questions that can arise in surrogacy, such as the payment of the surrogate. They also wanted to avoid an imbalance in genetic relatedness to the child, as one father in surrogacy families is genetically related to the child and the other is not. A study of gay pre-adoptive couples in the USA also found that men were concerned that surrogacy would be challenging due to the logistical and emotional challenges it can entail and considered this path to parenthood inaccessible due to its high cost (Goldberg *et al*., 2012).

For lesbian women and heterosexual couples who created their families using ART, a key motivation for doing so was the ability to have a genetic relationship with the child (Ragoné, 1994; Teman, 2010; Goldberg and Scheib, 2015). Lesbian mothers who started their families through donor insemination wanted to experience pregnancy and the birth of the child (Chabot and Ames, 2004; Lingiardi *et al*., 2016). The lesbian mothers in Goldberg and Scheib's (2015) study also had concerns about the cost and the complexity of adoption, the potential problematic background children may come from and the possibility of encountering discrimination and stigma during the adoption process.

Once intending gay couples have decided on surrogacy as a path to parenthood, they face a decision as to which father will have a genetic connection with the child. For those couples who have an equal desire for genetic parenthood, they can choose to mix their sperm together, or the eggs of a donor can be fertilized with sperm of both partners and multiple embryos can then be transferred to the surrogate (referred to as ‘intentional unknowing’) (Murphy, 2013). Other couples may choose to take genetic fatherhood in turns, with one partner providing sperm for the first child and their partner providing sperm for the following attempts. In making decisions about genetic fatherhood, intending fathers may also need to consider factors such as paternal age and the presence and heritability of health conditions (Greenfeld and Seli, 2011). How fathers think about these choices in retrospect is unknown.

Gay couples choosing surrogacy as a path to parenthood also have to consider whether to conceive a child through genetic or gestational surrogacy arrangements, both of which are currently practised in the USA. Little is known about men's motivations for the choices that they make. Although legislation differs across states, medical practitioners and agencies typically recommend gestational surrogacy as this arrangement gives intending fathers certainty over legal parentage (American Society for Reproductive Medicine, 2012). Some argue that there is a greater risk that surrogates who are genetically related to the child will change their minds about delivering the baby to the intending parents, although there is no empirical evidence to support this view (Imrie and Jadva, 2014).

Of all the ART available to intending parents, surrogacy is arguably the most controversial (Jadva, 2016). Only 19 states in the USA currently allow commercial gestational surrogacy to married same-sex couples and in 15 states it is practiced because no statute or published case law prohibits it (Creative Family Connection, 2016). Given its controversial nature and the fact that intending gay couples may live in a different state to the surrogate, support by families of origin (e.g. parents and siblings) and friends is important through the process and after the birth of the child (Hammarberg *et al*., 2015). A study of gay fathers of children born by surrogacy reported that the families of origin were supportive and excited to become grandparents, with the frequency of contact and visits increasing following the birth of children (Bergman *et al*., 2010). In donor insemination families headed by heterosexual couples and lesbian mothers, grandparents with a genetic connection to the child have been found to be more involved in the children's lives than grandparents who lack this connection (Fulcher *et al*., 2002). Whether families of origin react similarly to genetic fathers (whose parents and siblings will have a genetic connection to the child) versus non-genetic fathers (whose parents and siblings will not) is yet to be explored.

The present study explored gay fathers’ motivations for having a child through surrogacy and the various decisions involved in following this path to parenthood. Fathers’ feelings about these decisions were also examined. In addition, the study investigated how families of origin and friends responded to men becoming fathers in this way. Comparisons between genetic and non-genetic fathers were conducted in order to explore the relevance, or irrelevance, of genetic relatedness to fathers’ motivations for, and feelings about, surrogacy.

## Materials and Methods

### Participants

Data were collected as part of a larger investigation of parent–child relationships and child adjustment in gay father families formed through surrogacy (Golombok *et al*., 2016). A total of 40 families participated in the study, all of whom resided in the USA. The inclusion criteria for participation in the study were that the target child was aged 3–9 years old and that the parents had been a couple since the time of the child's birth. In this analysis, data were analysed from 74 fathers (in 6 families only 1 father was available for interview).

A variety of strategies were used to recruit as diverse a sample as possible. Firstly, surrogacy agencies that specialized in working with gay men sent information about the study to the fathers in their mailing list (*N* = 18, 45%); secondly, families were recruited at events at which gay fathers were in attendance (*N* = 15, 37.5%); thirdly, participants passed on information about the study to their friends, colleagues or acquaintances who fitted the study criteria and/or disseminated information about the study through social media (*N* = 7, 17.5%).

The mean age of the fathers was 47.29 years (SD = 6.20 years). The mean annual family income was $370 000 (SD = $168.264), which is unsurprising given the significant cost of commercial surrogacy arrangements in the USA. Most fathers were White (*N* = 67, 84%), with the remaining fathers identifying as Latino/Hispanic (*N* = 7, 9%), Asian (*N* = 1, 1%) or ‘other’ (*N* = 5, 6%). Ninety-eight percent of fathers had a Bachelor's or higher degree. Most families lived in the Northeast (67.5%; New York City, NY = 24, MA = 3), with the remaining families living in the South (7.5%; FL = 1, VA = 1, TX = 1), the West (22.5%; CA = 7, OR = 1, WA = 1) and the Midwest (2.5%; MN = 1).

Twenty-four (60%) of the target children were boys and 16 (40%) were girls, with an average age of 5.8 years (SD = 2.2 years). In most families (*N* = 28, 70%), the target child's siblings had been conceived through surrogacy, with one family (2.5%) having a child conceived in a previous heterosexual relationship and one family (2.5%) having an adopted child; in the remaining 10 families (25%), the target child had no siblings.

The majority of surrogacy arrangements (*N* = 38, 95%) were carried out in the USA with 2 (5%) conducted in India. Most surrogacy arrangements (*N* = 36, 90%) were gestational, with four couples (10%) conceiving through genetic surrogacy. In gestational surrogacy families, most surrogates (*N* = 35, 97%) were previously unknown to the couple and one surrogate (3%) was a friend. In families formed via genetic surrogacy, three surrogates (75%) were previously unknown to the couple and one was a sister of the non-genetic father (25%). In gestational arrangements, most parents (*N* = 34, 94%) had used a previously unknown egg donor, with one couple (3%) using a friend and one (3%) using the sister of the non-genetic father.

### Procedure

The majority of families (*N* = 26, 65%) were visited at home by a research psychologist trained in the study techniques. Due to geographical distance from the researchers, data were collected from the remaining families over Skype (*N* = 14, 35%). The fathers were presented with an information sheet and were given an opportunity to ask questions about the study before signing consent forms and participating in the study. Ethical approval was obtained from the University of Cambridge Psychology Research Ethics Committee and the New York State Psychiatric Institute Institutional Review Board.

### Measures

Of the 40 families who participated in the study, interviews were conducted with 74 fathers (in 6 families only 1 father was available for interview). Interviews lasting ~1 h were conducted with each father separately and a section of the interview focused on fathers’ experiences of surrogacy, using questions adapted from the UK longitudinal study of heterosexual families formed through surrogacy (MacCallum *et al*., 2003). This section of the interview was digitally recorded and transcribed verbatim.

Fathers were asked whether they and their partner had an equal desire to become parents; whether they had always preferred surrogacy or considered other paths to parenthood; why they had chosen surrogacy; the process of deciding whose sperm to use in the child's conception and why they had chosen to conceive a child through a gestational or genetic surrogacy arrangement. During this section of the interview, almost all of the fathers disclosed who the genetic father of the child was (if this information was known), with the exception of two fathers in one family. Fathers were also asked how they felt about having conceived a child through surrogacy, how they felt about their decision regarding genetic fatherhood and how their family and friends felt about their decision to become parents through surrogacy.

### Data analysis

Interviews were analysed using a text-driven, qualitative content analysis approach (Krippendorf, 2013), which aims to report participants’ motivations and experiences in as close a way as possible to their own interpretation (Sandelowski, 2000, 2010). This analytic approach was selected so that frequency counts could be calculated and group comparisons could be conducted.

The data were analysed by two coders, in a process comprising three stages: first, as the interviews were semi-structured, data of interest were dispersed throughout the transcript, therefore coder one organized the data into excel sheets (e.g. all quotations pertaining to ‘motivations for surrogacy’ were copied into one cell); second, coder one condensed the quotations in each excel cell into simple and succinct categories (e.g. ‘adoption less desirable path than surrogacy’), which comprised the first draft of the coding manual. Any questions or complexities that arose were discussed and agreed on between the first and second coder until a consensus was reached; third, using the coding manual, coder one analysed all of the data and one-third of the data were rated by coder two to calculate inter-rater reliability. Percentage agreement was equal or above 92% for each variable, rated as described below.

#### Choosing surrogacy as a path to parenthood

Desire for parenthood: genetic father more strongly; equal desire, non-genetic father more strongly; Preferred path to parenthood: always preferred surrogacy; considered adoption; attempted adoption; Motivations for surrogacy: adoption a less desirable path to parenthood than surrogacy (yes, no); desire for genetic relatedness with child as an individual or as a couple (yes, no); surrogacy was a financially viable option (yes, no); partner's choice (yes, no); desire for involvement in pregnancy and birth (yes, no); Decision about genetic parenthood: both donated sperm; one donated sperm-more important to one than the other; one donated sperm-turn taking; one donated sperm-medical reason; one donated sperm—sister as egg donor; Motivations for gestational surrogacy: prefer to separate genetic and gestational links to the child (yes, no); agency policies (yes, no), gestational surrogacy enabled a specific family set-up (yes, no).

#### Reflections on and reactions to surrogacy as a path to parenthood

Feelings about surrogacy: mostly satisfied, neutral, mostly dissatisfied; Feelings about whose sperm was used: mostly satisfied, neutral, mostly dissatisfied; Reactions of family of origin: mostly supportive, mixed, mostly negative; Reactions of friends: mostly supportive, mixed, mostly negative.

Data were obtained from 35 genetic fathers, 32 non-genetic fathers, 5 fathers who did not know the status of genetic parenthood in their family and 2 fathers who did know this information but chose not to disclose it during the interview. Chi-square tests were used to conduct comparisons between fathers’ parental status (genetic versus non-genetic) and quotations that illustrate the study findings are reported. Data were analysed using SPSS (IBM Corp., Armonk, NY, USA). A *P* < 0.05 was considered statistically significant.

## Results

### Choosing surrogacy as a path to parenthood

As shown in Table I, genetic and non-genetic fathers did not differ in their desire to become a parent (*χ*^2^ = 0.84 (2), *P* = 0.66). Likewise, there was no difference between genetic and non-genetic fathers in their preferred path to parenthood (*χ*^2^ = 3.43 (2), *P* = 0.18), with most fathers having considered adoption (*N* = 48, 65%), but only three fathers (4%) having attempted adoption as a path to parenthood.

**Table I:** Choosing surrogacy as a path to parenthood: gay fathers’ motivations and decisions.

	Genetic father (*N* = 35)	Non-genetic father (*N* = 32)	*χ*^2^	Do not know or disclose (*N* = 7)	Total (*N* = 74)
	*N* (%)	*N* (%)		*N* (%)	*N* (%)
*Desire for parenthood*			0.84 (2), *P* = 0.66		**(*N* = 67)**
Genetic parent more strongly	10 (28.5%)	6 (19%)		NA	16 (24%)
Equal desire	14 (40%)	13 (41%)		NA	27 (40%)
Non-genetic parent more strongly	10 (28.5%)	11 (34%)		NA	21 (31%)
Missing	1 (3%)	2 (6%)		NA	3 (5%)
*Preferred path to parenthood*			3.43 (2), *P* = 0.18		
Always preferred surrogacy	13 (37%)	8 (25%)		2 (29%)	23 (31%)
Considered adoption	20 (57%)	24 (75%)		4 (57%)	48 (65%)
Attempted adoption	2 (6%)			1 (14%)	3 (4%)
Motivations for surrogacy*					
Adoption a less desirable path to parenthood than surrogacy	23 (66%)	21 (66%)	*0.14 (1), P = 0.71*	6 (86%)	50 (68%)
Desire for genetic relatedness as an individual or couple	21 (60%)	13 (41%)	*1.79 (1), P = 0.18*	4 (57%)	38 (51%)
Surrogacy was a financially viable option	6 (17%)	4 (12.5%)	*0.18 (1),* *P* *= 0.67*	2 (29%)	12 (16%)
Partner's choice	3 (9%)	7 (22%)	*2.70 (1), P = 0.10*	0	10 (13%)
Desire for involvement in pregnancy and birth	1 (3%)	1 (3%)	*0.01 (1), P = 0.93*	0	2 (3%)
*Decision about genetic parenthood*			1.17 (4), *P* = 0.88		
Both donated sperm	16 (46%)	15 (47%)		6 (86%)	37 (50%)
One donated sperm-more important to one than the other	8 (23%)	8 (25%)		0	16 (22%)
One donated sperm-turn taking	6 (17%)	3 (9%)		0	9 (12%)
One donated sperm-medical reasons	2 (6%)	1 (3%)		0	3 (4%)
One donated sperm-family structure (e.g. sister as egg donor)	2 (6%)	2 (6%)		0	4 (5%)
Missing	1 (3%)	3 (9%)		1 (14%)	5 (7%)
Motivations for gestational surrogacy*	*N* = 31	*N* = 28			(*N* = 66)
Prefer to separate genetic and gestational links to the child	17 (55%)	13 (46%)	0.70 (1), *P* = 0.40	3	33 (50%)
Agency policies	17 (55%)	13 (46%)	0.70 (1), *P* = 0.40	1	31 (47%)
Gestational surrogacy enabled a specific family set-up	1 (3%)	1 (4%)	0.01 (1), *P* = 0.95	1	3 (5%)
Missing	7 (23%)	6 (21%)		0	13 (20%)
*Not applicable (genetic surrogacy chosen)*	*4*	*4*			*8*

*Some fathers described more than one motivation, thus percentages do not equal 100.

The most common reason given for pursuing surrogacy, given by approximately two-thirds of fathers (*N* = 50, 68%), was that adoption was a less desirable and/or achievable path to parenthood than surrogacy. As shown in the quotations in Table II, fathers felt that they would have had less control over the process of both becoming a parent and raising the child if they were to adopt.

**Table II:** Choosing surrogacy as a path to parenthood: illustrative quotations.

*Motivations for surrogacy*	
Adoption a less desirable path to parenthood than surrogacy	‘We liked surrogacy really because what we had read about adoption it seemed like quite a random process, and you weren't in control. Even after the child was born, there were all sorts of stipulations and criteria by which you, for no reason of your own, lose your child. And we just thought let's keep it simple, it's complicated enough being a parent.’ (Genetic father)
Desire for genetic relatedness with child	‘I guess we felt that we really wanted to have our own biological children as much as possible so we could possibly understand them more. In retrospect that's kind of a naive, ridiculous notion because I see how it is being a parent and having an adopted child is completely one hundred percent as satisfying as having a child through surrogacy, I now believe. At the time I didn't.’ (Genetic father)
Surrogacy was a financially viable option	‘And we had, the most important thing is that, at the time, we had enough money to do it. We don't anymore. But it is expensive, you know. You have to have $100,000 sitting around that you have no use for.’ (Non-genetic father)
Partner's choice	‘The primary reason is that [partner] wanted to have a child through surrogacy, and so it became quickly apparent to me that essential a condition, one of the non-flexible conditions of being in relationship with him, was that if we were going to have children it was going to be through surrogacy.’ (Genetic father)
Desire for involvement in pregnancy and birth	‘And I think we both felt like, number one, we really wanted to be part of the whole birth process.’ (non-genetic father)
*Decision about genetic parenthood*	
Both donated sperm	‘They're hard questions, it took a while. So we came up with this plan which we were going to use, some embryos would be from me, some embryos would be from him, so we had this great, neat plan. Then we got twins.’ (Genetic father)
One donated sperm-more important to one than the other	‘It just seemed inherently so much more important to [husband] and children were from his perspective, an important thing that he wanted and I loved him and wanted him to be happy. So it just seemed more logical that we would do that and I think that there was also that his parents were into the surrogacy and the genetic link was super important too and it wasn't that important to me.’ (Non-genetic father)
One donated sperm-turn taking	‘When we decided we were going to do surrogacy I wanted him to be the biological father of the girls. And then when we decided that we were going to have a third child it just, it just seemed natural to alternate, so I did; I was the biological father.’ (Genetic father)
One donated sperm-medical reasons	‘I have an aunt who was mentally disabled, and also like I'm older. There is talk about older fathers, autism being more prevalent.’ (Non-genetic father)
*Motivations for gestational surrogacy*	
Prefer to separate genetic and gestational links to the child	‘We thought just legally and emotionally it would be the best so that if you know we thought that it would be healthier for our relationship with the surrogate and healthier for the kids relationship with her because you know we were always very careful to say this is your surrogate you know, this is not your mother, we explained that to friends, because it's not her genetic egg it really isn't their mother and so we wanted that sense of separation.’ (Father didn't disclose genetic parenthood status)
Agency policies	‘Our surrogacy agency and our fertility clinic would only work with gestational carriers which is a separate egg donor and a separate gestational carrier so you have an agreement with both and both of them disavow their parental rights so it's just cleaner legally.’ (Genetic father)

The second most common response as to why fathers chose surrogacy was that they wanted to have a genetic connection with their child as this was important to them either as an individual, or as a couple (*N* = 38, 51%). Some fathers (*N* = 12, 16%) chose surrogacy because it was a financially viable option for them. Other fathers described becoming a parent through surrogacy because it was their partner's preference (*N* = 10, 13%). Lastly, two fathers (3%) pursued surrogacy because they wanted to be involved in the pregnancy and birth of the child. Differences between genetic and non-genetic fathers’ motivations for surrogacy were not statistically significant.

After deciding to pursue surrogacy as a path to parenthood, couples were faced with the decision as to which intending father would have a genetic link to the child. Half of the fathers in the study (*N* = 37, 50%) decided that they and their partner would both donate sperm, therefore leaving genetic parenthood to chance. In the remaining families, only one intending father donated sperm. In some cases, genetic parenthood was more important to one intending father than the other (*N* = 16, 22%); in others, the fathers had agreed to take genetic fatherhood in turns (*N* = 9, 12%); for three fathers (4%) medical reasons determined who should donate, and for four fathers, only one father donated because they had decided to use a sister as an egg donor (*N* = 4, 5%).

Another decision couples had to make was whether to choose a gestational or a genetic surrogacy arrangement, although the overwhelming majority opted for the former (36 out of 40 families). Of the 66 fathers who had conceived a child through gestational surrogacy, half chose to do so because they felt that there was a greater risk of an unsuccessful surrogacy arrangement if the surrogate had both a gestational and genetic link to the child (*N* = 33, 50%). The second most popular motivation for choosing gestational arrangements, once again endorsed by approximately half of the fathers, was because this was recommended to them by their surrogacy agency (*N* = 31, 47%).

### Reflections on and reactions to surrogacy as a path to parenthood

As shown in Table III, genetic and non-genetic fathers did not differ in how they felt about having conceived a child through surrogacy (*χ*^2^ = 0.43 (2), *P* = 0.81). Most fathers (*N* = 55, 74%) were satisfied with the surrogacy journey. Five fathers (7%) had neutral feelings about surrogacy. Five fathers (7%) were mostly dissatisfied. Fathers who were dissatisfied described the surrogacy process as a huge undertaking, which could produce feelings of anxiety and concern. Illustrative quotations of father's comments are shown in Table IV.

**Table III:** Reflections on and reactions to surrogacy as a path to parenthood.

	Genetic father (*n* = 35)	Non-genetic father (*n* = 32)	*χ*^2^	Do not know or disclose (*n* = 7)	Total (*n* = 74)
*Feelings about surrogacy*	*N* (%)	*N* (%)	0.43 (2), *P* = 0.81	*N* (%)	*N* (%)
Mostly satisfied	26 (74%)	23 (72%)		6 (86%)	55 (74%)
Neutral	2 (6%)	3 (9%)		0	5 (7%)
Mostly dissatisfied	3 (9%)	2 (6%)		0	5 (7%)
Missing	4 (11%)	4 (13%)		1 (14%)	9 (12%)
*Feelings about whose sperm was used*			1.10 (2), *P* = 0.58		
Satisfied	16 (45%)	12 (38%)		2 (29%)	30 (41%)
Neutral	8 (23%)	11 (34%)		2 (29%)	21 (28%)
Unsatisfied	1 (3%)	2 (6%)		0	3 (4%)
Not applicable (sister was egg donor)	2 (6%)	2 (6%)		0	4 (5%)
Missing	8 (23%)	5 (16%)		3 (42%)	16 (22%)
*Reaction of family of origin*			0.38 (1), *P* = 0.54		
Supportive	24 (68.5%)	19 (60%)		4 (57%)	47 (64%)
Mixed	10 (28.5%)	11 (34%)		2 (29%)	23 (31%)
Negative	0	0		0	0
Missing	1 (3%)	2 (6%)		1 (14%)	4 (5%)
*Reaction of friends*			0.03 (1), *P* = 0.87		
Supportive	31 (89%)	27 (84%)		5 (72%)	63 (85%)
Mixed	3 (8%)	3 (10%)		1 (14%)	7 (10%)
Negative	0	0		0	0
Missing	1 (3%)	2 (6%)		1 (14%)	4 (5%)

**Table IV:** Reflections on and reactions to surrogacy as a path to parenthood: illustrative quotations.

*Feelings about surrogacy*	
Mostly satisfied	‘For us it was, we just lucked out, it was such a wonderful process for us, and I'm sure it's not for other people, and I'm sure it's hard for other people to go through the process and not get pregnant and all that… It would be hard to have anything negative to say about the process at all. For us, other than the fact that it cost a lot of money, it was all wonderful…’ (Non-genetic father)
Neutral	‘I don't really think about it more anymore, it's just sort of like, I don't know, I just never even think, like I never even think about the fact that we're in a same-sex couple, I don't even think about the fact that we had them in an unconventional way. I think we lived in a region where people were constantly asking us about it, maybe, you know, I don't really think about it.’ (Non-genetic father)
Mostly dissatisfied	‘I thought it was painful, arduous for us personally, I think it could be a lot less if things that happened to us hadn't happened to us. So I think it's person dependant, I think it's experience dependant.’ (Genetic father)
*Feelings about whose sperm was used*	
Mostly satisfied	‘I love it. I couldn't imagine being closer to [child's name] if he was biologically my son.’ (Non-genetic father, satisfied)
Neutral	‘It's not something we give a whole lot of thought to.’ (Non-genetic father, neutral)
Mostly unsatisfied	‘It's very painful for me as somebody that didn't even want to have children, let alone bio children, this makes no sense at all but it hurts my spirit that neither one are mine. I can't help that.’ (Non-genetic father, unsatisfied)
*Reaction of family of origin*	
Mostly supportive	‘Well, I mean my father was unconditionally supportive, he was thrilled that we were going to have children, he felt as though it was the thing he had hoped for me and now it was happening, he paid for the cost of doing it very generously, offered to do that and then did that, so he was terrific.’ (Genetic father)
Mixed	‘They just had a lot, my family, my parents, had a lot of questions. They didn't understand at all. And it was, it was a little annoying actually I remember because I thought they were just going to be you know unbelievably excited, and instead of unbelievable excitement it was… I would probably characterise the reaction as confused, and a bit tentative, like we were doing some sort of crazy science experiment, and did we really understand what we were doing and was this a good idea… Yeah they were concerned and confused at first, and the unbelievable excitement eventually set in for them.’ (Genetic father)
*Reaction of friends*	
Mostly supportive	They were all very supportive, and happy for us, and loving (non-genetic father)
Mixed	‘They were very excited and supportive I would say, with the one possible exception of our gay friends. We didn't, and we don't, have a lot of gay friends, but the ones who we were friends with at the time, these were the same people who when [partner] and I decided to get married they were, they seemed to be the least excited, as though we were somehow changing our lives to conform to society's norms in a way that they didn't think was you know necessary… So ironically it was our gay friends who I would say, it wasn't that they weren't, but they certainly seemed the least excited about what we were doing.’ (Genetic father)

When asked how they felt about their decision on whose sperm would be used to conceive their child, the responses of genetic and non-genetic fathers did not differ (*χ*^2^ = 1.10 (2), *P* = 0.58). Most fathers felt either satisfied (*N* = 31, 42%) or neutral (*N* = 21, 28%), with just three fathers (4%) feeling mostly dissatisfied with their decision.

The fathers were asked how their families of origin reacted to them becoming parents. Family members’ reactions were no different for genetic compared to non-genetic fathers (*χ*^2^ = 0.38 (1), *P* = 0.54). Most fathers (*N* = 47, 64%) described their family's reactions as supportive. Approximately one-third (*N* = 23, 31%) of fathers reported that their family's reaction was mixed. Fathers described their parents and/or siblings as having questions or feeling confused, but then embracing the child when he or she finally arrived.

As for the reactions of friends, once again there was no difference in the reports of genetic and non-genetic fathers (*χ*^2^ = 0.03 (1), *P* = 0.87). The majority of the fathers reported that this was positive (*N* = 63, 85%), although some did note that the quality and nature of some friendships changed once they became a parent. A minority of fathers (*N* = 7, 10%) reported that their friends’ reactions were mixed, with some fathers describing less support from gay than heterosexual friends, or friends and colleagues having questions about the ethical aspects of surrogacy.

## Discussion

The findings of this study indicate that a number of factors influence men's decisions to choose surrogacy as a path to parenthood. The most popular reason given by the fathers in the study was that they felt that adoption was a less desirable and/or accessible path to parenthood. Some fathers explained that at the time that they were thinking of becoming a parent, adoption was prohibited to same-sex couples, or that open-adoption was the only option permitted to them. Other fathers felt that a child adopted by same-sex parents may be subjected to greater levels of stigma. Until the June 2015 Supreme Court decision regarding marriage equality (Obergefell versus Hodges), same-sex couples and single lesbian and gay people could not legally adopt children in some states in the USA (American Psychological Association, 2015). Eventhough legislation has changed, legal ambiguities remain about second-parent adoptions by lesbian and gay partners and the rights of unmarried same-sex couples to access domestic adoption (Smalling, 2016) and experiences of discrimination from child welfare agencies are not unusual (Downing *et al*., 2009; Goldberg *et al*., 2012). It is therefore unsurprising that these factors affect men's decisions as they consider their options on how to start their families.

Whilst the ability to have a genetic connection to their child was of great value to some gay fathers, it was not important to all. Approximately half of the fathers (*N* = 38, 51%) described having chosen surrogacy as a path to parenthood so that they themselves could have a genetic connection to the child, or because surrogacy allowed them as a couple to have a genetic tie to the child. For one-fifth of families in the study (*N* = 16, 22%), only one father donated sperm to create embryos. The fathers in these families described genetic fatherhood as only important to one parent, rather than to both of them. The findings of the present study are in contrast to a Spanish study of 10 gay father surrogacy families, in which fathers did not state a strong belief in the importance of genetic ties (Smietana *et al*., 2014). The culture and socio-legal constraints in the countries in which gay men raise their families may be an important factor in shaping intending fathers’ beliefs about the significance of genetic relatedness for family relationships. Whereas surrogacy is one of the most regulated paths to parenthood through ART in the USA (Perkins *et al*., 2016), it is prohibited in Spain and its use is controversial (Smietana *et al*., 2014).

The findings of the study help to shed light on surrogacy practices in the USA. Fathers chose gestational surrogacy arrangements because they did not want the surrogate to have both a genetic and gestational connection to the child. This is consistent with the finding that for some (but not all) gay fathers, the genetic link between that father and the child was considered to be significant. Whereas this connection was desirable for themselves as fathers, the men in the study wanted to avoid the risk that there would be a bond between the surrogate and the child. A similar proportion of fathers reported being influenced by the agencies with whom they worked, who typically favoured gestational over genetic surrogacy arrangements. Therefore, decisions about the surrogacy process appear to be shaped not only by men's beliefs about the significance of genetic relatedness but also by agency policies, which are arguably driven and no doubt formed to some degree by state legislation.

The majority of fathers were satisfied with having chosen to have a child through surrogacy, and with their choice about who would be the genetic father. However, owing to the use of a volunteer sample in this study, the possibility that those fathers who had a particularly positive experience were more likely to participate in the research cannot be ruled out. Future research would benefit from a longitudinal approach, which would avoid the biases inherent in retrospective recall. Another challenge is to acknowledge and appreciate the sensitivity of exploring personal issues such as the significance of genetic relatedness for family relationships. The interviewers were trained to be aware of the potential sensitivity of questions pertaining to genetic fatherhood, and in some interviews, these questions were not asked, at the interviewer's discretion. Questions were also omitted if a child or family member interrupted the interview, or due to time constraints.

Consistent with previous studies of gay fathers in surrogacy families (Bergman *et al*., 2010), the majority of the families received support from their families and friends when they became parents through surrogacy. However, for one-third of the gay fathers (*N* = 23, 31%), family members were initially confused or perplexed about the surrogacy process, but then supportive once the child arrived. As the mean age of the fathers was 47 years and surrogacy has only recently become a viable route to parenthood (Perkins *et al*., 2016), especially for gay men, these reactions from family members and friends are perhaps unsurprising.

Genetic and non-genetic fathers did not differ in their reports of their family's reactions to them becoming parents through surrogacy. The quality of relationships between gay fathers and their children in surrogacy families and their grandparents, aunts and uncles has not been examined. As gay fathers in surrogacy families can and do take turns in being the genetic father, their families of origin may have a genetic link to at least one child in the family. Fathers may also have chosen not to disclose the identity of the genetic father to their children, extended family, friends or acquaintances (Dempsey, 2013; Murphy, 2013; Carone *et al*., 2016) and therefore the status of genetic relatedness may be unknown.

This study contributes to the small yet growing literature that explores the significance, or insignificance, of genetic relatedness for gay father surrogacy families. Understanding the motivations or gay men and their experiences of both genetic and non-genetic fatherhood may be helpful for clinicians, practitioners and counsellors working with this population. The more that is known about this growing family type, the better informed policy and practice can be.
